# Next generation genome sequencing reveals phylogenetic clades with different level of virulence among *Salmonella* Typhimurium clinical human isolates in Hong Kong

**DOI:** 10.1186/s12864-015-1900-y

**Published:** 2015-09-14

**Authors:** Chi Keung Cheng, Man Kit Cheung, Wenyan Nong, Patrick Tik Wan Law, Jing Qin, Julia Mei-Lun Ling, Kai Man Kam, William Man Wai Cheung, Hoi Shan Kwan

**Affiliations:** School of Life Sciences, The Chinese University of Hong Kong, Hong Kong SAR, China; Department of Microbiology, The Chinese University of Hong Kong, Prince of Wales Hospital, Hong Kong SAR, China; Centre for Health Protection, Department of Health, Hong Kong SAR, China; Current address: Stanley Ho Centre for Emerging Infectious Diseases, JC School of Public Health, Faculty of Medicine, The Chinese University of Hong Kong, Hong Kong SAR, China

**Keywords:** Foodborne infection, Epidemiology, Phylogeny, Virulence determinants, Single nucleotide polymorphism, Antimicrobial susceptibility

## Abstract

**Background:**

*Salmonella* Typhimurium is frequently isolated from foodborne infection cases in Hong Kong, but the lack of genome sequences has hindered in-depth epidemiological and phylogenetic studies. In this study, we sought to reconstruct the phylogenetic relationship and investigate the distribution and mutation patterns of virulence determinants among local *S.* Typhimurium clinical isolates using their genome sequences.

**Results:**

We obtained genome sequences of 20 *S*. Typhimurium clinical isolates from a local hospital cluster using a 454 GS FLX Titanium sequencing platform. Phylogenetic analysis was performed based on single nucleotide polymorphism positions of the core genome against the reference strain LT2. Antimicrobial susceptibility was determined using minimal inhibitory concentration for five antimicrobial agents and analyses of virulence determinants were performed through referencing to various databases. Through phylogenetic analysis, we revealed two distinct clades of *S*. Typhimurium isolates and three outliers in Hong Kong, which differ remarkably in antimicrobial susceptibility and presentation and mutations of virulence determinants. The local isolates were not closely related to many of the previously sequenced *S*. Typhimurium isolates, except LT2. As the isolates in the two clades spanned over 10 years of isolation, they probably represent endemic strains. The outliers are possibly introduced from outside of Hong Kong. The close relatedness of members in one of the clades to LT2 and the Japanese stool isolate T000240 suggests the potential reemergence of LT2 progeny in regions nearby.

**Conclusions:**

Our study demonstrated the utility of next-generation sequencing coupled to traditional microbiological testing method in a retrospective epidemiological study involving multiple clinical isolates. The evolution of multidrug- and ciprofloxacin-resistant strains among the more virulent clade is also an increasing concern.

**Electronic supplementary material:**

The online version of this article (doi:10.1186/s12864-015-1900-y) contains supplementary material, which is available to authorized users.

## Background

*Salmonella* foodborne infection is a common but important public health issue worldwide. Among the many serovars, *Salmonella* Typhimurium is frequently isolated from outbreaks as one of the common bacterial causative agents. The World Health Organization has also emphasized the rising concern of multidrug resistance in this non-typhoid *Salmonella* serovar, which potentially accounts for the transfer of antimicrobial resistance to other human pathogens [1]. With the continual reduction in the cost for high-throughput genome sequencing, thousands of genomes of pathogenic bacteria have now been sequenced and *Salmonella* is of no exception [2]. In addition to the conventional analysis of antimicrobial resistance profiles, coupling of genome sequencing to phylogenetic analysis has opened new trends of in-depth epidemiological studies at both regional and global levels. Over the past few years, hundreds of genomes of various *Salmonella* serovars, including Typhimurium [3], Enteritidis [4], Typhi [5], Newport [6], Heidelberg [7], and Pullorum [8], were sequenced to facilitate evolutionary studies, as well as epidemiological and pathogenicity investigations in this important pathogen.

Despite the availability of genome sequences for *S*. Typhimurium isolates from all around the world, the Japanese strain T000240 remained as the only sequenced and published isolate from northeastern Asia [9]. Here we report the use of high-throughput genome sequencing, coupled to traditional microbiological testing method, in a retrospective study of *Salmonella* Typhimurium strains isolated from subjects hospitalized in Hong Kong over the past two decades. Specifically, we reconstructed the phylogenetic relationship and investigated the distribution and mutation patterns of virulence determinants among 20 local isolates.

## Methods

### Bacterial strains

A total of 20 *S.* Typhimurium isolates (Table 1) were obtained from patients admitted to the hospitals of the New Territories East Cluster of the Hospital Authority in Hong Kong between 1993 and 2007. Written informed consent for using the blood and stool samples in the study was obtained from all participants. Seven blood isolates, three of which isolated in the mid 90’s and the rest isolated in the mid 00’s, and 13 stool isolates were obtained by standard procedures. The blood isolates are representatives of the circulating clones during the sampling periods and act as representatives of systemic infection whereas the stool isolates were used as the genetic background for comparison purpose. The 10-year span between isolate collections allows determination of endemicity of the selected strains. These isolates were confirmed biochemically by the AP120E system (bioMérieux S.A., Montalieu Vercieu, France).

**Table 1:** Information of patients and corresponding *S*. Typhimurium clinical isolates

Isolate	Source	Year of isolation	Patient age	Patient sex
ST728/07	Blood	2007	2	F
ST4024/07	Blood	2007	54	M
ST4848/06	Blood	2006	68	M
ST2850/05	Blood	2005	81	M
ST4650/95	Blood	1995	4	M
ST6988/94	Blood	1994	78	F
ST8493/93	Blood	1993	14	F
ST372/06	Stool	2006	15	M
ST1660/06	Stool	2006	8	M
ST2286/06	Stool	2006	1	F
ST486/06	Stool	2006	8	F
ST2533/06	Stool	2006	18	M
ST1489/06	Stool	2006	28	F
ST4650/06	Stool	2006	5	F
ST2143/05	Stool	2005	2	F
ST4329/05	Stool	2005	1	F
ST4038/02	Stool	2002	37	M
ST3363/96	Stool	1996	7	M
ST3858/96	Stool	1996	43	F
ST2287/95	Stool	1995	11	F

### Genome sequencing and *de novo* assembly

Genomic DNA from the isolates was extracted using PrepMan Ultra Reagent (Applied Biosystems) according to the manufacturer’s instructions. Whole-genome shotgun sequencing was performed on a 454 GS FLX Titanium platform (Roche Diagnostics) [10]. Bases sequenced and corresponding quality values were called and delivered in standard format by GS FLX for downstream bioinformatic analyses. Sequence reads were assembled *de novo* using Newbler assembler (Roche Diagnostics).

### SNPs extraction and phylogenetic analysis

All SNP positions were obtained by aligning the genome sequences of the 20 isolates with the reference strain LT2 [11] chromosome using Mauve and 454 GS Reference Mapper [10]. Raw SNP calls were filtered to ensure that at least 90 % of the reads support the SNP. SNPs called in phage sequences and repetitive regions of the reference genome were excluded. Only SNPs located in the *Salmonella* core genes [12] were included in the phylogenetic analysis. All remaining SNPs were concatenated to generate a single pseudo-sequence. Phylogenetic analyses were conducted in MEGA (version 5.21) [13] and phylogenetic trees were reconstructed using the Maximum Parsimony (MP) method with a heuristic search based on the Tree Bisection and Reconnection (TBR) approach. *Salmonella* Enteritidis PT4 (GenBank Accession AM933172) and *Salmonella* Choleraesuis SC-B67 (GenBank Accession AE017220) were used as outgroups. Nodal supports were inferred from 500 bootstrap replicates.

### Antimicrobials resistance profiling

The 20 *S*. Typhimurium isolates were tested for susceptibility to ampicillin, gentamicin, chloramphenicol, trimethoprim, and ciprofloxacin by an agar dilution method according to the recommendations of the Clinical and Laboratory Standards Institute (CLSI) [14]. Isolates with minimal inhibitory concentrations (MICs) greater than those for susceptible strains as suggested by CLSI were regarded as resistant. Multidrug resistance was defined as resistant to three or more of the antimicrobials tested.

### Virulence determinants analysis

Genes and mutations responsible for antimicrobial resistance were retrieved from the literature and compared among the 20 isolates. Virulence factors and *Salmonella* Pathogenicity Islands (SPIs) for *Salmonella* pathogenicity were obtained from the Virulence Factors Database (VFDB) (http://www.mgc.ac.cn/VFs/) and aligned against each of the respective genome sequences for the detection of genetic variations [15]. Prophage elements for the isolates were identified by the web server PHAge Search Tool (PHAST) (http://phast.wishartlab.com/) [16].

## Results

### Phylogenetic tree analysis revealed two major phylogenetic clades in Hong Kong

**Fig. 1:**
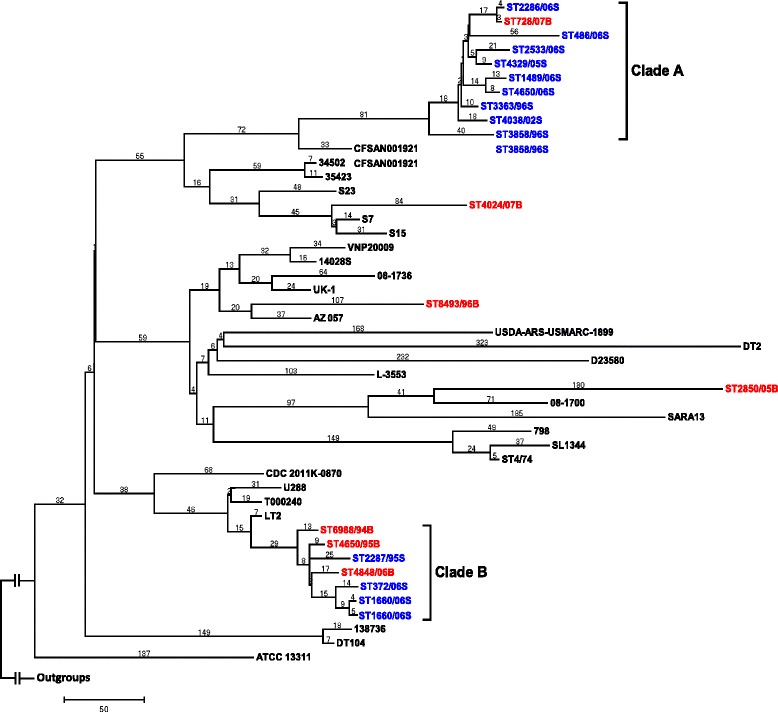
Maximum-parsimony phylogenetic tree of 47 *S.* Typhimurium genomes based on SNPs identified by mapping to the LT2 reference genome. Only SNPs in the “core” genes were included. The tree was rooted using *Salmonella* Enteritidis PT4 (GenBank Accession AM933172) and *Salmonella* Choleraesuis SC-B67 (GenBank Accession AE017220). Red isolates: local blood isolates; Blue isolates: local stool isolates; Black isolates: reference GenBank isolates. The number on each branch is the number of SNP differences. The scale bar represents the number of SNPs

Genomes of 20 local *S.* Typhimurium isolates were sequenced here with an average depth of 38× (Table 2). The SNP-based phylogenetic trees grouped the *S*. Typhimurium isolates into two major phylogenetic clades (Fig. 1, Additional file 1, Additional file 2 and Additional file 3). Clade A consisted of 10 isolates with a predominance of nine stool isolates and only a single blood isolate, whereas clade B consisted of a total of seven isolates including three blood isolates and four stool isolates. The remaining three isolates appeared to be sporadic infections and they were also distantly related by themselves. Intriguingly, they were all blood isolates. The year of isolation did not seem to be an important determining factor in the phylogeny, as isolates retrieved from the 90’s and 00’s were both found in each of the clades.

**Table 2:** Statistics for the 20 sequenced *S.* Typhimurium genomes

Isolate	Total length (bp)	Read no.	Contig no.	N50 (bp)	Fold coverage	GenBank accession
ST728/07	4,674,705	587,022	51	297,458	54	JRYT00000000
ST4024/07	4,710,407	410,923	42	324,707	38	JRYU00000000
ST4848/06	4,835,948	1,116,351	33	412,176	87	AUXE00000000
ST2850/05	4,823,833	370,563	49	324,996	33	JRZV00000000
ST4650/95	4,839,422	364,948	32	413,043	31	JRZX00000000
ST6988/94	4,821,910	570,727	49	311,185	47	JRZW00000000
ST8493/93	4,821,249	214,096	58	149,902	18	JRZU00000000
ST372/06	4,812,346	158,272	82	100,492	14	JRZT00000000
ST1660/06	4,817,227	689,231	33	412,269	55	JRZS00000000
ST2286/06	4,680,742	274,344	64	226,047	25	JRZR00000000
ST486/06	4,694,852	797,428	90	297,457	71	JRZQ00000000
ST2533/06	4,671,245	209,116	64	197,512	18	JRZP00000000
ST1489/06	4,667,695	240,650	57	225,759	18	JRZO00000000
ST4650/06	4,667,933	230,841	56	226,048	17	JRZN00000000
ST2143/05	4,856,278	775,034	37	458,267	68	JRZM00000000
ST4329/05	4,667,023	216,066	59	192,711	16	JRZL00000000
ST4038/02	4,675,722	301,882	59	223,187	25	JRZK00000000
ST3363/96	4,675,601	653,493	46	324,537	53	JRZJ00000000
ST3858/96	4,672,420	341,172	45	423,111	32	JRZI00000000
ST2287/95	4,846,386	371,971	36	412,238	33	JRZH00000000

### Contrasting antimicrobials resistance profiles among phylogenetic clades

The 20 isolates were tested for their susceptibility to five antimicrobials from different classes (Table 3). Fifteen of the isolates (75 %) were resistant to at least one antimicrobial class. More than half of the strains were resistant to ampicillin (70 %), trimethoprim (60 %) and chloramphenicol (55 %), while less was resistant to ciprofloxacin (25 %) and gentamicin (20 %). Except gentamicin, the proportion of resistance to ampicillin, trimethoprim, chloramphenicol, and ciprofloxacin was higher in the blood isolates (100, 86, 71 and 29 %) than the stool isolates (54, 46, 46 and 23 %). Among the 20 isolates, 11 (55 %) were multidrug-resistant, in which five were blood isolates. Intriguingly, with respect to the phylogenetic tree above, all seven isolates within clade B were multidrug-resistant, with all of them resistant to the older antimicrobials ampicillin and chloramphenicol and six of them resistant to trimethoprim. In contrast, among the 10 isolates from clade A, only two of them were multidrug-resistant, with at least half of them still susceptible to these older antimicrobials.

**Table 3:** Susceptibility to five antimicrobials for the 20 *S.* Typhimurium isolates

Isolate	Source	Clade	Year of isolation	AMP	GEN	CHL	TRI	CIP	Total # of resistance
ST728/07	Blood	A	2007	R	S	S	R	S	2
ST4024/07	Blood	--	2007	R	S	R	R	R	4
ST4848/06	Blood	B	2006	R	R	R	R	R	5
ST2850/05	Blood	--	2005	R	S	S	S	S	1
ST4650/95	Blood	B	1995	R	S	R	R	S	3
ST6988/94	Blood	B	1994	R	S	R	R	S	3
ST8493/93	Blood	--	1993	R	S	R	R	S	3
ST372/06	Stool	B	2006	R	R	R	R	R	5
ST1660/06	Stool	B	2006	R	S	R	R	R	4
ST2286/06	Stool	A	2006	R	S	S	R	S	2
ST486/06	Stool	A	2006	S	S	S	S	S	0
ST2533/06	Stool	A	2006	R	R	S	R	S	3
ST1489/06	Stool	A	2006	S	S	R	S	S	1
ST4650/06	Stool	A	2006	S	S	S	S	S	0
ST2143/05	Stool	B	2005	R	S	R	S	R	3
ST4329/05	Stool	A	2005	S	S	S	S	S	0
ST4038/02	Stool	A	2002	S	S	S	S	S	0
ST3363/96	Stool	A	1996	R	S	R	R	S	3
ST3858/96	Stool	A	1996	S	S	S	S	S	0
ST2287/95	Stool	B	1995	R	R	R	R	S	4

*AMP* Ampicillin, *GEN* Gentamicin, *CHL* Chloramphenicol, *TRI* Trimethoprim, *CIP* Ciprofloxacin

### Loss of virulence determinants in clade A isolates

Genome sequence analysis revealed the absence of the virulence plasmid pSLT, a ~90 kb plasmid of LT2 which harbors many important virulence factors including the *spv* locus, *pef* (plasmid-encoded fimbriae) locus and the complement resistance gene *rck* [17], in the 10 clade A isolates.

The *Salmonella* Pathogenicity Islands (SPIs), which encode two type III secretion systems (T3SS) and a number of virulence effectors, represent another category of important virulence factors. Genome sequences revealed that SPI1-5 were present in all 20 isolates and were highly conserved in sequence. However, a number of SNPs were found in SPIs in isolates from clade A, for instance, *fhlA* (nucleotide position 1916) in SPI1, *orf242* (pos. 541) and *sseC* (pos. 1272) in SPI2 as well as *sugR* (pos. 183) and *mgtB* (pos. 351) in SPI3 (Additional file 4). The SNP in *sseC*, which was shown to be an important effector protein to alter host cell physiology and promote bacterial survival [18], resulted in a previously undescribed Glu424 > Asp amino acid change. Another effector protein sseI/srfH, which lies within the Gifsy-2 prophage, also showed a SNP at nucleotide position 139 and resulted in a Ala47 > Thr amino acid change. Nevertheless, other effector proteins, including those encoded outside of SPI1 and SPI2 such as *sopB* and *sopE2*, did not show any sequence variation.

Apart from the pSLT plasmid and SNPs in the SPIs, isolates in clade A also contained less genetic materials arisen from prophages. All isolates from clade B contained a complete copy of the *Salmonella* prophages Gifsy-1 and Gifsy-2 [19, 20], whereas isolates from clade A contained only ~39 and ~68 % genetic materials from the respective prophages. This apparent reduction of genomic content had resulted in the loss of several genes previously implicated to involve in long-term systemic infection in mice (STM2585, 2586, 2596, 2597, 2635 [Gifsy-1]) and replication in macrophages (STM1031, 1033, 1041 [Gifsy-1], 2585, 2589, 2595, 2599, 2603, 2605 [Gifsy-2]) [21, 22]. While five out of the seven isolates from clade B contained a complete copy of Fels-2, isolates from clade A and the three sporadic isolates did not harbor this *Salmonella* prophage. In addition, isolates in clade A had also lost a total of ~20 % of genetic materials from phage ST104 compared to clade B isolates, whereas sporadic isolates ST2850/05 and ST8493/93 did not harbor ST104. Instead, these two isolates contained a complete copy of the phage ST64B, which is also identified in many previously sequenced *Salmonella* isolates but not in isolates in clade A, B, LT2 and the Japanese isolate T000240 [9].

## Discussion

### The potential reemergence of LT2 progeny

*Salmonella* Typhimurium is one of the most common bacterial causes of foodborne infections in Hong Kong, with 150–200 reported cases each year. Nevertheless, genomes of these clinical isolates have seldom been sequenced. In this report, we present genome sequences of 20 *S*. Typhimurium clinical isolates in Hong Kong throughout 1993–2007. Phylogenetic analysis indicated that two major phylogenetic clades (represented by clade A and B in Fig. 1) had been circulating in Hong Kong for almost the past two decades, with some sporadic infections caused by phylogenetically distinct isolates. We showed that several of the previously sequenced *S*. Typhimurium isolates, including the human isolates DT104 [23] and D23580 [24], did not show high phylogenetic relatedness to isolates either in clade A or B (Fig. 1). Notably, isolates from clade B showed remarkable genetic relatedness to the laboratory reference strain LT2, which was originally isolated in the 1940s. Comparative genomic analysis also indicated that the Japanese isolate T000240 displays high similarity to isolates in clade B. Izumiya et al. [9] commented that multidrug-resistant progeny of LT2 might be reemerging alongside DT104 and other definitive phage-type strains, and our data suggested that such progeny of LT2 might have already reemerged in regions nearby Japan over at least the past two decades.

### Analysis of antimicrobial resistance determinants

**Fig. 2:**
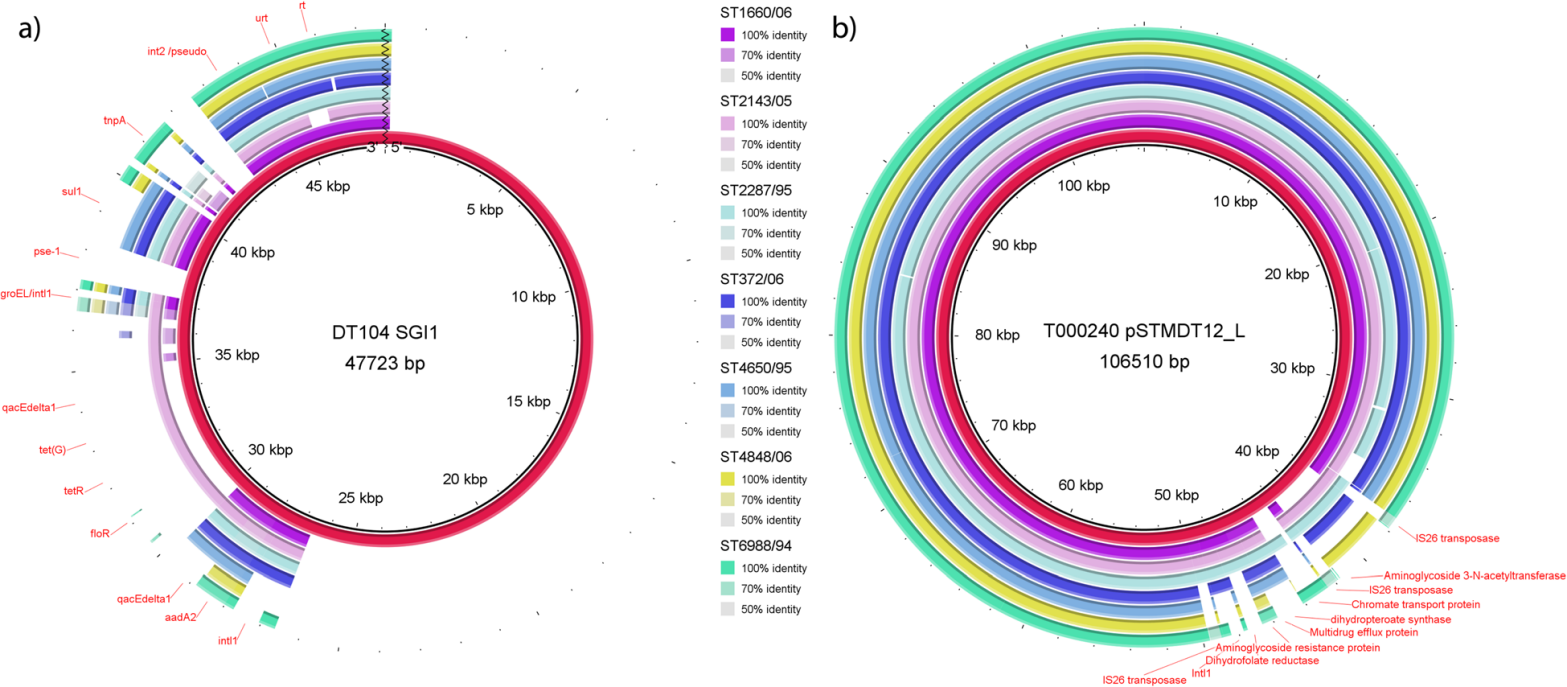
BRIG diagrams showing an overview of the genomic relationship between the seven sequenced group B *S.* Typhimurium isolates and **a** the SGI1 genomic island in DT104 (GenBank Accession AF261825) and **b** the T000240 plasmid pSTMDT12_L (GenBank Accession NC_016861) [29]. The innermost rings (*in red*) represent the reference sequences, and the outer rings show BLASTN comparisons of the group B genomes against the references using an E-value cut-off of 0.001. Known genes of SGI1 in DT104 [25] and known protein products of open reading frames in the resistance island of pSTMDT12_L [9] are marked on the outermost rings in (**a**) and (**b**), respectively

We also showed that the clade A and B isolates differed remarkably in their level of virulence in terms of antimicrobials resistance, presence of virulence plasmids and prophage elements. Not only did clade B comprise a higher proportion of blood isolates, all isolates within the clade were also multidrug-resistant. Genome sequence alignment revealed that none of our 20 local isolates harbor a complete copy of the *Salmonella* Genomic Island 1 (SGI1) found in the DT104 lineage [25]. Specifically, all isolates in clade A did not harbor any of the genomic fragments from SGI1. Intriguingly, isolates in clade B harbor an approximately 5.2 kb-fragment originated from SGI1 (Fig. 2a), which is represented by a class 1 integron consisting of OXA-1 beta-lactamase *bla*_oxa-30_, aminoglycoside resistance protein *aadA1*, a small multidrug resistance protein *qacEΔ1* and sulfonamide resistance gene *sul1*. This class 1 integron is in turn located within the previously characterized 82 kb GI-DT12 genomic island in T000240. Together with the chloramphenicol acetyltransferase and tetracycline resistance protein *tetA* genes [26] located 6.7 and 10.8 kb upstream, respectively, to the class 1 integron, this genomic island confers resistance to a number of antimicrobials including ampicillin, kanamycin, chloramphenicol, tetracycline, sulfonamide drugs, and quaternary ammonium compounds.

The large plasmid pSTMDT12_L identified in T000240 is also exclusively found in the clade B isolates (Fig. 2b). Nevertheless, due to the presence of four transposases (three of which are IS26) and a recombination protein in the plasmid, the resistance island showed structural variation, which results in the existence of the aminoglycoside 3-N-acetyltransferase gene (*aac (3)*, for gentamicin resistance) in only four of the isolates and the dihydrofolate reductase gene (*dfrA1*, for trimethoprim resistance) in only three of the isolates.

Ciprofloxacin resistance in *Salmonella* Typhimurium as well as other serovars, most notably Typhi and Paratyphi, has become a global concern in recent years [27]. Five out of our 20 isolates (ST4806/06, ST372/06, ST1660/06, ST2143/05, and ST4024/07) were shown to be ciprofloxacin-resistant. The first four isolates were from clade B, and they demonstrated similar mutation patterns in the quinolone resistance-determining regions (QRDRs) in the DNA gyrase A (*gyrA*) and DNA topoisomerase IV subunit A and B (*parC* and *parE*) genes [28]. Genomic sequences revealed a Ser83 > Phe mutation in *gyrA* for all the four isolates, but at amino acid 87, it was Asp87 > Asn for ST4806/06 and Asp87 > Gly for the remaining three. Mutation for *parC* was a consistent Ser80 > Arg, but for the *parE* gene it was the rarely described Leu416 > Phe for ST4806/06 and the more common Ser458 > Pro for the rest. No mutations were identified in the *gyrB* gene. Interestingly, the only ciprofloxacin-resistant strain outside clade B, ST4024/07, showed only a single mutation (Asp87 > Tyr) without any additional mutation in either *gyrB*, *parC* or *parE* genes. This suggests that only a single mutation in the QRDR of *gyrA* is sufficient to confer resistance to ciprofloxacin.

### Evolution of ciprofloxacin-resistant strains

As noted above, ciprofloxacin resistance was only identified in strains isolated in the 00’s and not noted in the 90’s. In particular, four of these isolates were from the more virulent clade B. Despite additional antimicrobials has not been tested, resistance to ciprofloxacin has often been associated with quinolones resistance. Such combination of multidrug and a potential quinolone resistance has prompted clinicians to pay attention to the spread of progenies from *S*. Typhimurium strains in clade B.

## Conclusions

Our study revealed the existence of two major phylogenetic clades of *Salmonella* Typhimurium clinical isolates circulating in Hong Kong over the past two decades. The two clades differ remarkably in antimicrobial susceptibility, presentation and mutations of virulence determinants and members in one of the clades are shown to be close relatives and likely progenies of the laboratory reference strain LT2. Such potential dissemination of this multidrug-resistant group of *S*. Typhimurium in the northeast Asia should deserve more attention.

### Availability of supporting data

The whole genome shotgun data sets generated in this study have been deposited at DDBJ/EMBL/GenBank under the accessions JRYT00000000, JRYU00000000, AUXE00000000, JRZV00000000, JRZX00000000, JRZW00000000, JRZU00000000, JRZT00000000, JZS00000000, JRZR00000000, JRZQ00000000, JRZP00000000, JRZO00000000, JRZN00000000, JRZM00000000, JRZL00000000, JRZK00000000, JRZJ00000000, JRZI00000000, and JRZH00000000.

## Additional files

Additional file 1:
**Maximum-parsimony phylogenetic tree of 47** ***S.*** **Typhimurium genomes with bootstrap values reported on nodes.** Only SNPs in the “core” genes were included. The tree was rooted using *Salmonella* Enteritidis PT4 (GenBank Accession AM933172) and *Salmonella* Choleraesuis SC-B67 (GenBank Accession AE017220). Red isolates: local blood isolates; Blue isolates: local stool isolates; Black isolates: reference GenBank isolates. The number at each node is the support value inferred from 500 bootstrap replicates. Bootstrap values <50 are not shown here. The scale bar represents the number of SNPs. (PDF 93 kb) Additional file 2:
**Maximum-parsimony phylogenetic tree of 92** ***S.*** **Typhimurium genomes based on SNPs identified by mapping to the LT2 reference genome.** Only SNPs in the “core” genes were included. The tree was rooted using *Salmonella* Enteritidis PT4 (GenBank Accession AM933172) and *Salmonella* Choleraesuis SC-B67 (GenBank Accession AE017220). Red isolates: local blood isolates; Blue isolates: local stool isolates; Black isolates: reference GenBank isolates. The number on each branch is the number of SNP differences. The scale bar represents the number of SNPs. (PDF 40 kb) Additional file 3:
**Maximum-parsimony phylogenetic tree of 92** ***S.*** **Typhimurium genomes with bootstrap values reported on nodes.** Only SNPs in the “core” genes were included. The tree was rooted using *Salmonella* Enteritidis PT4 (GenBank Accession AM933172) and *Salmonella* Choleraesuis SC-B67 (GenBank Accession AE017220). Red isolates: local blood isolates; Blue isolates: local stool isolates; Black isolates: reference GenBank isolates. The number at each node is the support value inferred from 500 bootstrap replicates. Bootstrap values <50 are not shown here. The scale bar represents the number of SNPs. (PDF 23 kb) Additional file 4:
**SNPs located at virulence genes of** ***S.*** **Typhimurium.** The list of *S.* Typhimurium virulence genes was retrieved from VFDB. Nucleotide positions were based on the LT2 reference genome. (XLSX 54 kb)
