# Supplementary figures and images for: Next generation genome sequencing reveals phylogenetic clades with different level of virulence among Salmonella Typhimurium clinical human isolates in Hong Kong
Source: BMC Genomics. 2015 Sep 14;16(1):688. doi: 10.1186/s12864-015-1900-y (PMC4570558; doi:10.1186/s12864-015-1900-y)

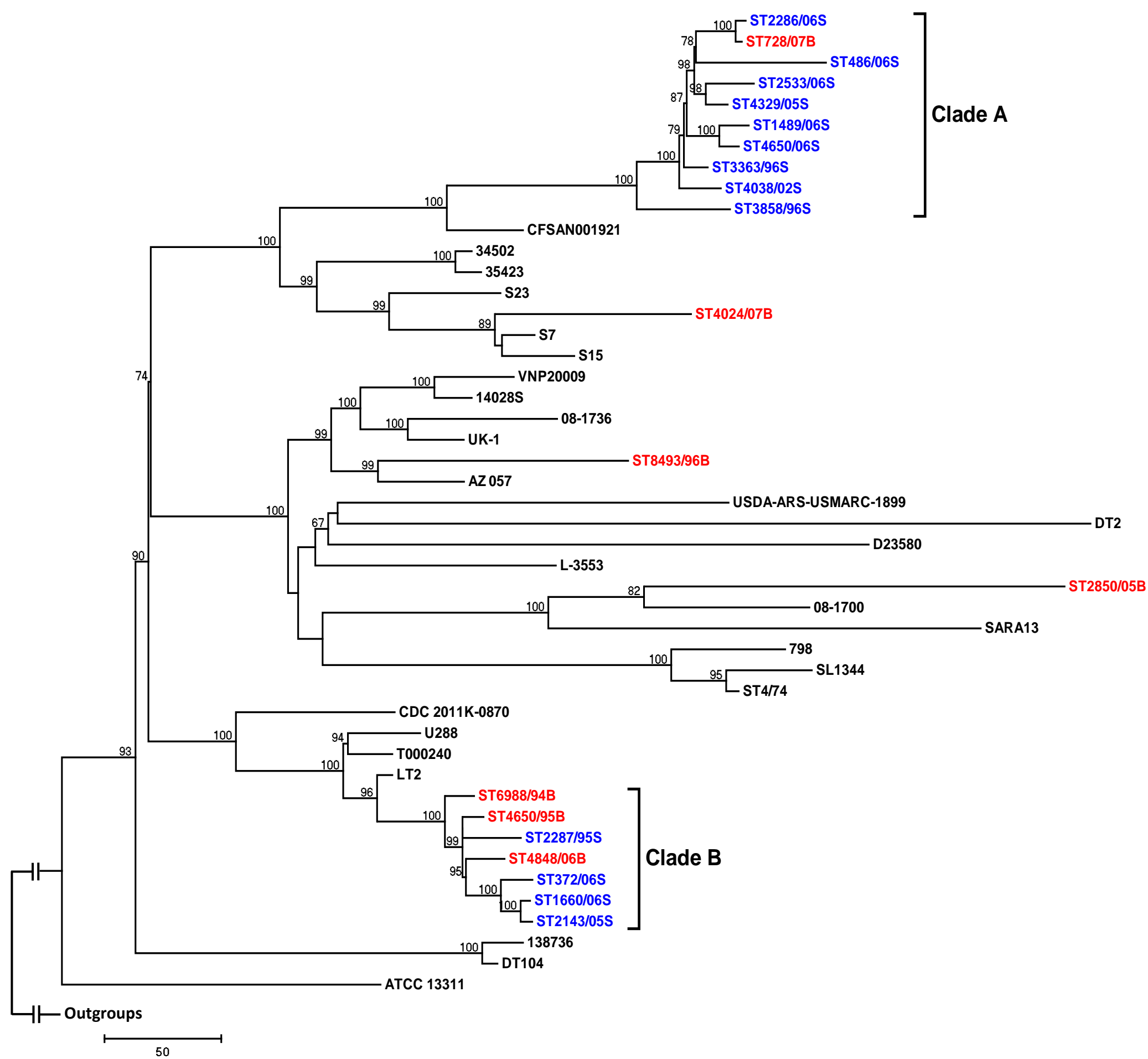

Supplement: Additional file 1: — Maximum-parsimony phylogenetic tree of 47 S. Typhimurium genomes with bootstrap values reported on nodes. Only SNPs in the “core” genes were included. The tree was rooted using Salmonella Enteritidis PT4 (GenBank Accession AM933172) and Salmonella Choleraesuis SC-B67 (GenBank Accession AE017220). Red isolates: local blood isolates; Blue isolates: local stool isolates; Black isolates: reference GenBank isolates. The number at each node is the support value inferred from 500 bootstrap replicates. Bootstrap values <50 are not shown here. The scale bar represents the number of SNPs. (PDF 93 kb) [file 12864_2015_1900_MOESM1_ESM.pdf]

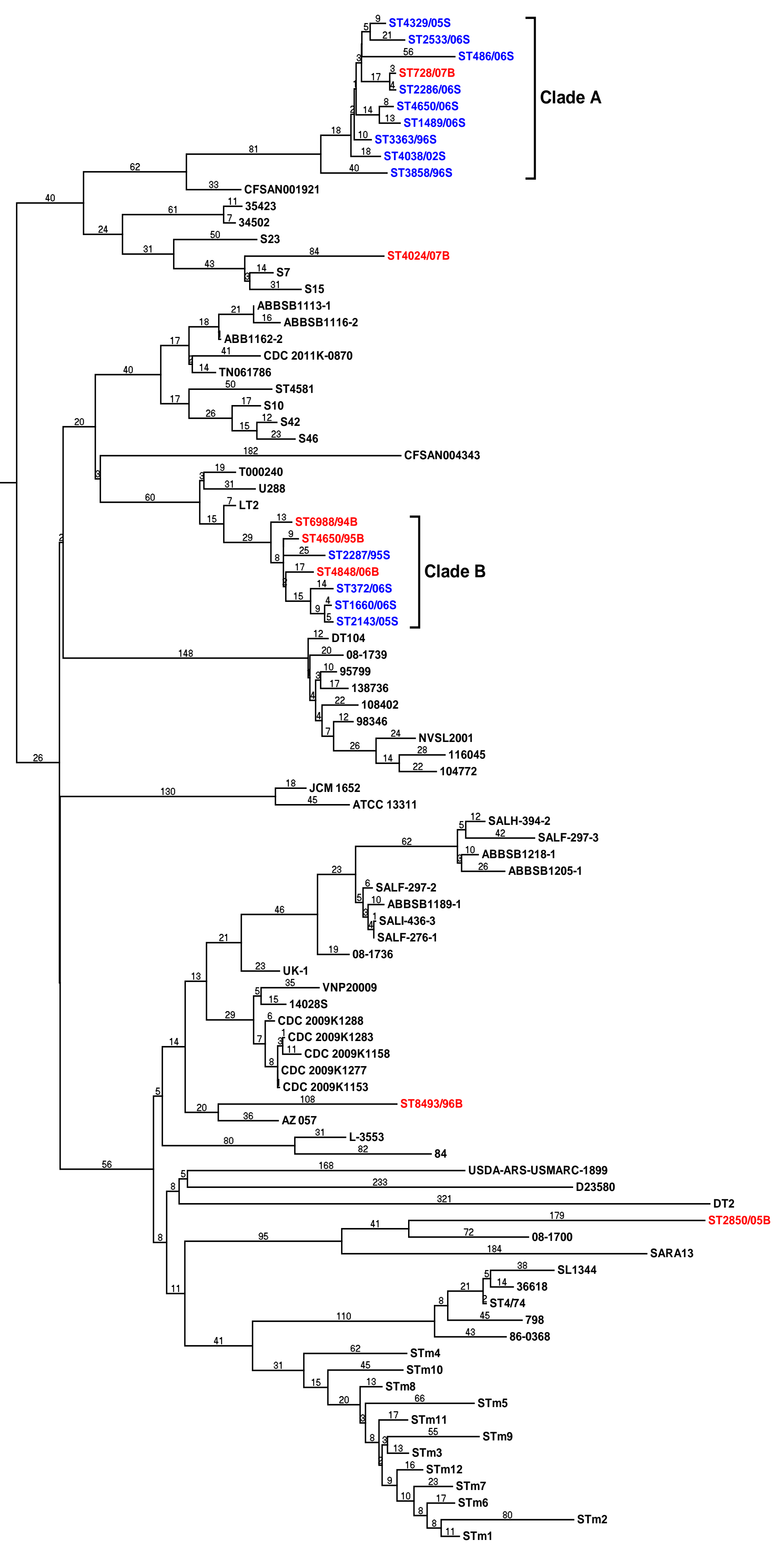

50

Supplement: Additional file 2: — Maximum-parsimony phylogenetic tree of 92 S. Typhimurium genomes based on SNPs identified by mapping to the LT2 reference genome. Only SNPs in the “core” genes were included. The tree was rooted using Salmonella Enteritidis PT4 (GenBank Accession AM933172) and Salmonella Choleraesuis SC-B67 (GenBank Accession AE017220). Red isolates: local blood isolates; Blue isolates: local stool isolates; Black isolates: reference GenBank isolates. The number on each branch is the number of SNP differences. The scale bar represents the number of SNPs. (PDF 40 kb) [file 12864_2015_1900_MOESM2_ESM.pdf]

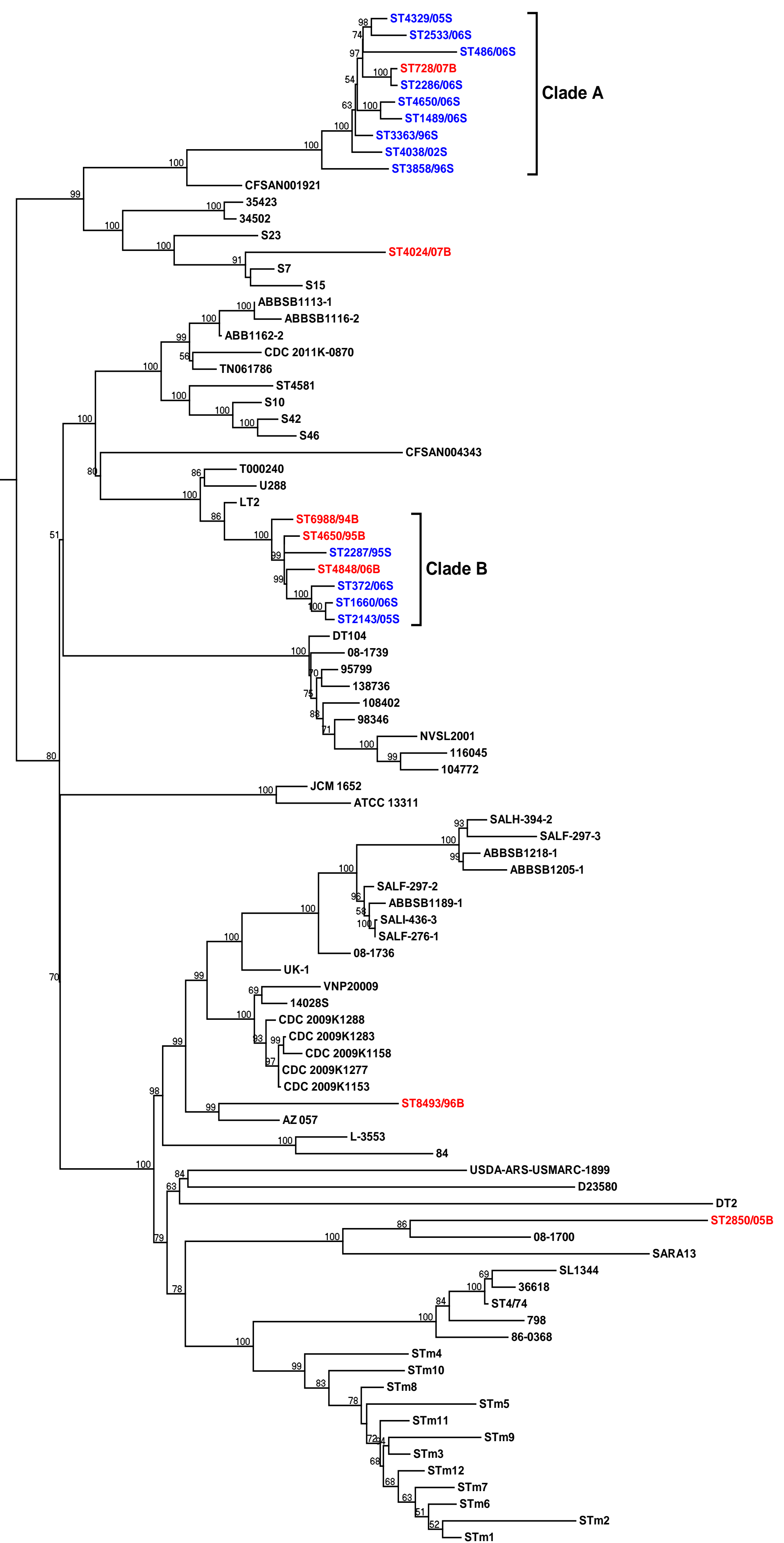

50

Supplement: Additional file 3: — Maximum-parsimony phylogenetic tree of 92 S. Typhimurium genomes with bootstrap values reported on nodes. Only SNPs in the “core” genes were included. The tree was rooted using Salmonella Enteritidis PT4 (GenBank Accession AM933172) and Salmonella Choleraesuis SC-B67 (GenBank Accession AE017220). Red isolates: local blood isolates; Blue isolates: local stool isolates; Black isolates: reference GenBank isolates. The number at each node is the support value inferred from 500 bootstrap replicates. Bootstrap values <50 are not shown here. The scale bar represents the number of SNPs. (PDF 23 kb) [file 12864_2015_1900_MOESM3_ESM.pdf]
